# "Is it just so my right?" Women repossessing breastfeeding

**DOI:** 10.1186/1746-4358-3-12

**Published:** 2008-08-04

**Authors:** Paige Hall Smith

**Affiliations:** 1 Center for Women's Health and Wellness, and Department of Public Health Education, School of Health and Human Performance, University of North Carolina at Greensboro, Greensboro, North Carolina, USA

## Abstract

Infant feeding occurs in the context of continued gender inequities and in the context of a feminist movement that left women vulnerable to a system that defined the male body and mind as the norm. This paper draws from a qualitative analysis of interviews conducted with women artists at the 2005 Mamapalooza music festival in New York City, and conference participants at the 2005 La Leche League International and International Lactation Consultant Association Conferences and at the 2007 Reproductive Freedom Conference to understand our collective alienation from breastfeeding and to outline a process for how we might repossess breastfeeding as a positive function in women's lives. These women find power in honoring and validating their own experiences, in claiming those experiences as legitimate feminist actions, and then drawing on these experiences to seek new meanings, customs and norms that similarly honor, value and support their rights to those experiences. They argue that we need a feminist movement that fully incorporates women's needs as biological and reproductive social beings, alongside their needs as productive beings, and a movement that defines the female body and mind as the norm.

## Background

Infant feeding occurs in the context of other continued gender inequities that include: lack of support by family, worksites and communities for breastfeeding; the sexualization and objectification of women's breasts; public aversion to the "maternal breast" which stigmatizes public breastfeeding and limits women's mobility in public spaces; lack of third party health insurance coverage for breastfeeding support; fragmentation of health care (obstetric care separate from pediatrics) and health care providers' failure to adequately inform women about the benefits of breastfeeding. All of these help create an environment that medicalizes and commercializes infant feeding while undermining women's capacity to breastfeed and alienating them from their maternal breast. These issues are visited even more heavily on low income and minority women as is demonstrated in shorter breastfeeding duration [1].

## Methods

Over the last couple of years, I have conducted interviews with women to identify the values that women bring to their decision-making about how to allocate their time, their money, their energy and their bodies in meeting their needs as individuals, workers and mothers; how women today manage their own efforts to achieve personal fulfillment and economic independence alongside their goals as mothers [2]; and then to use these women's experiences to develop a framework for feminist breastfeeding promotion. My purpose was *not *to assess the average or typical view of all women; rather I sought to interview women gathered around shared values, concerns or experiences. To date, I have sought out women gathered around the power of motherhood, breastfeeding advocacy and feminism.

This paper draws from analysis of interviews conducted with women artists at the 2005 Mamapalooza music festival in New York City (power of motherhood; 12 participants, "Mamapalooza"), and conference participants at the 2005 La Leche League International and International Lactation Consultant Association Conferences (breastfeeding advocacy; 11 participants, "LLLI/ILCA") [both in Washington, D.C.] and at the 2007 Reproductive Freedom Conference held by Hampshire College in Amherst, MA. (feminism; 6 participants, "Reproductive Freedom"). Open-ended interviews lasted between 30 and 45 minutes and were guided by questions designed to engage women in a discussion of their experiences with motherhood, breastfeeding, employment, the (dis)connections between breastfeeding, motherhood and feminism, and strategies for improving women's lives to make it easier for them to be employed and achieve personal fulfillment while still meeting their goals as mothers and ensuring the wellbeing of their children. The interviews were transcribed and entered into Atlas ti, a software for analysis of text based data and thematic analysis was used. This paper examines these women's experiences with breastfeeding and motherhood to understand our collective alienation from breastfeeding and to outline a process for how we might repossess breastfeeding as a positive function in women's lives.

Ethical approval for this research was granted by the Institutional Review Board of the University of North Carolina, Greensboro. All intended participants were provided with a consent form and they all consented.

### Conceptual framework

Repossession is a strategy commonly used by women and other groups who have experienced oppression as a way of reconnecting to previously alienated parts of their bodies, experiences and lives. For over 30 years women have been marching together at night through red-light districts and other dangerous parts of cities as a way of reclaiming our right to walk at night. The popular *Vagina Monologues *provides a way for women to reclaim and revalue vaginas [3]. Matria and Mullen based on their study of women reclaiming menstruation, conceptualized a three step process to repossession that emerges out of a sense of alienation from parts of ourselves, our bodies and our lives: reconnection; redefining; and normalizing [4]. According to Matria and Mullen, reconnection starts with women getting in touch with and validating personal experiences, unlearning concealment, sorting out what to keep, and filling in the gaps [4]. Redefining draws on personal experience to reinterpret old mythology, and substitutes positive explanations for negative ones. Step three, "normalizing", refers to trying out new perspectives and behaviors beyond immediate networks and seeks to normalize breastfeeding for society at large. Normalizing comes from creating customs and norms that increase women's comfort with themselves and each other, helping them live as they choose. This conceptualization provided a framework for examining women's repossession of breastfeeding as normal and healthy.

## Results

Themes emerged for each of the four components of the framework (Alienation, Reconnection, Redefining, Normalizing); these are presented below with supporting quotes.

### Alienation from breastfeeding

With some exceptions, this group of women was not alienated from motherhood or breastfeeding. Nonetheless, they put forward several ideas that help shed light on how, from their point of view, liberal feminist theory and praxis may have contributed to women's alienation from breastfeeding. These themes, with quotes illustrating some of them, are below:

• Feminism stressed the goal of helping women become players in a man's game in a man's world

"The . . . first wave of . . . feminism . . . defines feminism in terms of [women] taking on the men's roles . . . played with their rules . . . and denying your reproductive role . . . You know, we consider equality that we make ourselves like them and be accepted like them, the men. And not in the sense that we have a right to make the game be played the way it is comfortable for us." (LLLI/ILCA)

• Feminism feared biology becoming women's destiny

". . . feminism in the '70s . . . needed to remove the biology of motherhood . . . I think [if I had seen] breastfeeding . . . the pregnant body as something beautiful it would have helped me, . . . 14 years later . . . I'm still feeling divorced somewhat from the experience . . . I was so shut down, turned off and depressed and not really allowed to have those experiences . . . I feel a little cheated". (Mamapalooza)

• Feminism was afraid of disrespecting women who do not breastfeed

"I feel like part of the reason feminists are shying away from [breastfeeding] is that, 'Well, we already got into trouble for all the other shit we stirred up with the good mother, bad mother, stay-at-home versus go to work, do this, do that' . . ." (Reproductive Freedom)

• Feminism formulated a narrow construction of reproductive rights (limited to contraception and abortion)

"I highly protest when people say [breastfeeding] is not [a reproductive right] because I think that you cannot detach it from the whole experience of reproduction at all, because it is that natural cycle of what's supposed to happen, and I think that one of the problems is that we do not talk about it as part of that whole feminist model of abortion rights, conceptive, pregnancies, safe childbirth, breastfeeding. Breastfeeding seems like a natural end, but a lot of people want to cut it off which is very surprising to me because I mean [feminism] is a realm that is used to dealing with controversy; so, I cannot believe that breastfeeding would be that controversial that they would not want to deal with it." (Reproductive Freedom)

### Reconnection

**Figure 1:**
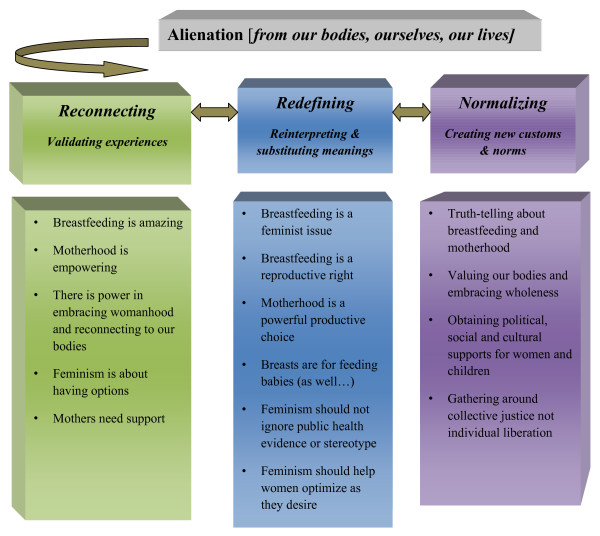
Repossessing breastfeeding: Reconnecting, redefining, and normalizing.

Figure 1 illustrates a three step iterative process of women's repossession of breastfeeding that emerged from this study. Step 1 in the Repossession process is Reconnection, which involves getting in touch with and validating with our own experiences. The following themes emerged from across the interviews:

• Breastfeeding is amazing and empowering

"And I think the best thing I've ever done in my life is breastfeed my children. It was the most empowering feeling thing I have ever experienced as a woman. Absolutely. And we have this miracle fluid that comes through our bodies . . . It's a miracle tissue. It's a miracle." (LLLI/ILCA)

• Mothering is empowering

" . . . I was never dying to have children . . . But . . . when my son was born I felt like I had walked into another dimension of life. And all these people were on the other side of his crib saying, "See, get it?" And I walked into this other part of living and went, "Oh, I get it. This is what it's all about." And the experience of parenting and mothering is so deep and so extraordinary if you allow it to be . . . You know, blowing the walls off your life and allowing yourself to learn all the things that come to you. I mean I've learned so much about myself . . . And just everything that your child teaches you . . . So, as far as an empowering thing if you allow it to enter your life as a character builder, yeah, it's amazing. It's incredible." (Mamapalooza)

• There is power in embracing the whole of womanhood and reconnecting to our biology

"I'm not going to work full-time and put my kids in daycare. I'm going to stay home. I'm going to be a mom. I'm going to embrace motherhood. We're actually doing just as much to further the feminist movement . . . The feminist movement is about being feminine. And what is more feminine than raising a baby? I mean that's what we are here for really. And I think we've kind of lost sight of that. I mean biologically we wouldn't be here as a species if we didn't reproduce . . . I think . . . the pendulum swung so far to say to be a feminist is to deny the traditional role of a woman instead of embracing all the roles that women can have. You know? I'm not saying that I didn't enjoy being in the Army. I didn't enjoy being a soldier. I was pretty good at it. I liked it." (LLLI/ILCA)

• Feminism is having options and making educated choices

"I definitely [consider myself to be a feminist]. And a lot of feminists may disagree because . . . I am home with my daughter and even before . . . I was using natural family planning . . . And I'm breastfeeding an extended period of time which a lot of feminist women I know would not consider to be feminist because they would consider I'm giving up a portion of myself for an extended period of time. But I actually feel that it's the opposite. It's a feminist act because I choose to do it willingly because I know the options that are available to me . . . and I feel that's what makes a woman feminist. Not the specific decision that she makes but having all the options and making an educated choice . . . I am in control of my body in a natural way which means that I don't have a physician or a pharmaceutical company telling me what I need to take or what I need to do with my body. (LLLI/ILCA)

• Mothers need and deserve support for breastfeeding, especially in public spaces including work

"And, so, I would go early in the morning . . . I'd go to the gym. I'd shower. I'd change. I would go to the daycare center and nurse my son. And then I would go to class. And then at lunch I would leave and go back to the daycare center and nurse. And the daycare center people told me, 'You're not allowed to eat in the lounge because we can't have babies in the staff lounge . . . No, you can't eat in the nursery; it's against the regulations for health code and stuff.' They said, 'Why don't you eat in the car? . . .' I said, '. . . I'm not going to go eat in my car. I take my lunch break to spend time with my son.' And I said, 'I can't possibly be the only person who's ever requested to eat lunch with their child.' And they said, 'Actually you are.' (LLLI/ILCA)

### Redefining

Step 2 in the Repossession process is Redefining: this involves using personal experience to reinterpret myths, stereotypes and norms and substitute positive explanations for negative ones. The women I interviewed were revaluing and redefining many different things including how they understood breastfeeding, motherhood, feminism and women's reproductive rights.

• Breastfeeding is a feminist issue

Interviewer: "So, you see breastfeeding as a feminist issue?"

Respondent: "Oh, definitely. Definitely. I even got into a debate with a friend who is not a mom, is in graduate school. And I was talking about breastfeeding in public as a feminist issue. And she said, 'Oh, come on; don't even link those two together. There's no such thing. It's a public health issue, breastfeeding in public because of the exchange of bodily fluids.' And she didn't see it as when you make a mother feel uncomfortable about her choice to breastfeed out in public chances are she is going to be isolated. If she is isolated she will stop breastfeeding and then she won't get the benefits of breastfeeding for herself or her child. So, it really is a feminist issue because of women's health and children's health is a feminist issue". (LLLI/ILCA)

• Breastfeeding is a reproductive right

"We talk a lot about reproductive rights [at this conference] . . . We heard a lot about reproductive rights, and then that whole discussion was about midwifery and doulas and all surrounding birth, . . . – I felt like we were ending right there at birth; . . . sort of once the baby is born that's the end of it . . . Certainly breastfeeding plays into that in a huge way, because it affects, you know, whether women can go back to work at a certain – if you want to breastfeed and you have to go back to work full-time, I mean that impacts that decision, and that impacts the health of your child and whether financially how quickly you can get back in the workforce, and you have to compromise. I mean I think there's an awful lot of social implications surrounding that decision to breastfeed or not, and I think as a feminist issue, yeah." (Reproductive Freedom)

• Motherhood is a powerful, productive choice

"Being a mother has been the most developmentally important thing that has happened in my life I think probably . . . But I mean just unbelievable what it does. It changes you. I can see where someone might could find their music. I found – I found a different sense of heart and a depth of compassion that even as a nurse I don't think I had. It's fascinating. Kids do amazing things to you. They drive you nuts, too, while they're doing it. I don't want to paint a rosy picture. They absolutely drive you crazy, but we can tolerate it." (LLLI/ILCA)

• Breasts are for feeding babies (as well . . .)

"The radical feminism says there is something unique and intrinsically female that we need to honor and recognize . . . [And] when breastfeeding become really important to me, . . . I had a framework to fit that into. I'm not giving up feminism. I'm looking at radical feminism and what that feels like . . . So in terms of for myself using my body in the way that it's designed to be used. That was a very feminist act. In fact I had my daughter and I was in the computer lab in the civil engineering department and there was an ad for cast iron pipe and it was this really large breasted woman. I can't remember the slogan but it was – I remember it was cast iron pipe. And someone said something to me about breastfeeding my daughter in the computer lab. And I said, 'If that woman is using her breasts to sell cast iron pipe, I'm going to use my breast to feed my baby right here.' So, that was a very feminist act right there. So, I never felt like the two were in conflict." (LLLI/ILCA)

• Feminism should not buy into the formula culture, ignore public health evidence or stereotype women

"It makes me angry that women are turned off by learning more . . . about having a healthier birth, a balanced breastfeeding, maybe about other methods of birth control, about other choices that they have because of these negative stereotypes or myths that they hear about certain types of groups . . . [But] we do not all have to do it the same way . . . When you allow formula companies and pharmaceutical companies and doctors who are primarily male . . . to influence your choice, that's not feminist." (LLLI/ILCA)

• Feminism is about making it possible for women to optimize themselves as humans regardless of decisions they make about how to live their lives

"I think that it was completely natural that women stood up and rejected the traditional rules of motherhood that have been put upon us back then . . . Unfortunately, I think . . . the media and outside sort of twisted that and pitted women against each other. I think that a lot of times women were saying, I reject this brand of idea for myself, not I reject these women. But on the outside it may – it turned into some kind of like cat fight. Again, which is what constantly happens with feminism and when women speak up for themselves. [Motherhood and feminism] work very well together. I think a feminist mother is a woman who is complete – or has [by raising children] become self-realized. Not relying on the tenets and beliefs of the larger society but of those that she feels she holds dear to herself. So, it's not just about like raising your child." (Mamapalooza)

### Normalizing

The last step in the iterative Repossession process is Normalizing, which comes from trying out new customs outside one's own social group to create new norms that increase women's comfort with themselves and each other and which make our lives more possible. The Normalizing customs I derived from these interviews are:

• Truth-telling, about motherhood and breastfeeding: the good and the bad

*"I felt like a frigging goddess. I felt like – like that was true feminism. Well at first did not feel like a goddess. First I felt like a human sacrifice . . . And I'm propped up on pillows thinking she's eating me alive! . . . And, also, I thought it was amazing that I was keeping her alive . . . at first those two years I could not meditate. There was no quiet time to separate. And I did not want to separate. So, I knew that every couple of hours I was going to have 40 minutes of total silence. And, OK, so someone was eating me at the time. But, you know, I adjusted my meditation practice". (Mamapalooza*)

• Valuing our bodies and embracing wholeness

"I think feminist is womaness. And I think breastfeeding is the ultimate of being a woman . . . I mean then you are going to [have a] civil war within your own self . . . I do not see why you cannot be feminine and a woman and a feminist and support breastfeeding . . . it just does not seem like . . . they should be separated". (Mamapalooza)

• We need political, social and cultural supports for women and children

"We need to push in the political arena . . . we're not valuing families; we are not valuing children in our society . . ., and I think that it starts with the first step of valuing them from birth and saying 'When you have a baby if you need to return to your job economically after you have your baby, we need to make sure, ensure that that happens' . . . [We need more policies] like the Family Medical Leave Act . . . so that people aren't left with the Hopson's choice of 'I can either have a baby and parent it the way it needs to be parented or I can eat'." (LLLI/ILCA)

• We need to come together around collective justice not individual liberation

"I don't know that I'm a feminist. I'm a humanist. I'm a Beingist. I believe in equality, absolutely. But . . . the roots of traditional feminism were so born out of [the] upper middle class whites realm that really, . . . instead of raising themselves up with everyone else said, "I'm going to work so you can take care of my child now . . . I believe that there is a necessity for women to have all the rights that men have, all the pay that men have, all the freedoms that men have, absolutely . . . But, you know, I think that duality is a tricky thing. And to call something just feminism without saying it's a movement for people of color. It's a movement for . . . queer individuals. I mean it has to be all inclusive. It can't be women need this right but . . . we don't want to look at anybody else . . . I think that true feminism would be about being the experience of all aspects of womanhood and motherhood not just the ones that are profitable . . . [A]ny time you try to better yourself at the expense of others it's going to fail . . . [T]hat's the kind of movement that needs to be built, not just something just empowers women . . . But you can't empower women at the expense of their children. You can't empower women at the expense of their domestic helpers." (Mamapalooza)

## Conclusion

Hausman writes that "Breastfeeding provides a focus that encourages us to see women's bodies at the centre of the dilemmas of modern societies, as women are increasingly called to labor in ways that disturb or make impossible the biosocial practices of maternity" [5] (p. 283). One dilemma identified by the women interviewed here is that the feminist strategy of advocating for women's equality with men left women vulnerable to a system that, while making it possible for women to be more engaged in the labor force, still defined the male body and mind as the norm. They suggest that women's alienation from their own bodies and from motherhood, from the *feminine*, may hinder women's, in particular mother's, abilities to become fully human. Nonetheless, as feminists have done for decades, these women find power in honoring and validating their own experiences as breastfeeding mothers, in claiming those experiences as legitimate feminist actions, and then drawing on these experiences to seek new meanings, customs and norms that honor, value and support their rights to those experiences without otherwise excluding them from public opportunities and rewards. They argue that we need a feminist movement that fully incorporates women's needs as biological and reproductive social beings, alongside their needs as productive beings, and a movement that defines the female body and mind as the norm. Then, perhaps breastfeeding could become normalized as a labor of love.

## Competing interests

The author declares that they have no competing interests.
